# Description of a transient proximal tubulopathy induced by amino acids perfusion in peptide receptor radionuclide therapy: A case report

**DOI:** 10.1097/MD.0000000000018478

**Published:** 2019-12-27

**Authors:** Rémi Lenain, Aghilès Hamroun, Georges Lion, Paul Chamley, Linh Bui, Arnaud Lionet, Marc Hazzan, François Provôt

**Affiliations:** a Department of Nephrology; b Department of Nuclear Medicine, Centre Hospitalier Regional Universitaire de Lille, Lille, France; c Nephrology Department, Centre Hospitalier de Beuvry, Béthune, France.

**Keywords:** [177Lu]-DOTA0-Tyr3-octreotate, amino acids, peptide receptor radionuclide therapy, proximal tubulopathy

## Abstract

**Rationale:**

Peptide receptor radionuclide therapy (PRRT) with radiolabeled somatostatin analogs is a targeted internal radiotherapy method used to treat tumors expressing somatostatin receptors. Concomitant amino acids perfusion is systematically performed in order to inhibit the proximal tubular uptake of the radionuclide and thus prevent nephrotoxicity.

**Patient concerns:**

a 67-year-old woman with an intestinal neuroendocrine tumor with multiple lymphadenopathies and liver metastases. The patient displayed a carcinoid syndrome with flushes including facial erythrosis and paresthesia. During the treatment, the patient exhibited emesis and severe cramps.

**Diagnosis:**

We describe incomplete proximal tubulopathy induced by an amino acid therapy with [177Lu]-DOTA0-Tyr3-octreotate, which was reversible after treatment discontinuation. This diagnosis relies on metabolic acidosis, hypophosphatemia due to renal loss, tubular proteinuria and generalized aminoaciduria. Serum creatinine remained stable during and after the procedure.

**Interventions:**

PRRT with radiolabeled somatostatin analog ([177Lu]-DOTA0-Tyr3-octreotate). In order to prevent PRRT induced nephrotoxicity, we used a solution of 20 amino acids including 22 g/L Lysine and 16.8 g/L Arginine. Metoclopramide was successfully used to control vomiting. During the treatment and at the time of cramps, the blood sample showed hypophosphatemia at 0.3 mmol/L justifying intravenous phosphate supplementation. The cramps disappeared after this infusion.

**Outcomes:**

Hypophosphatemia with low TmPO4/GFR was observed as well as an increase in β2-microglobulinuria, urinary polyclonal light chains, and amino aciduria involving all amino acids. All these disturbances disappeared the day after the treatment and there was no acute kidney injury after 5 PRRT sessions. Six months after PRRT discontinuation, the patient had neither renal failure nor proximal tubulopathy. Aminoacid induced tubulopathy involves the main ligands of the megalin receptor. It has recently been demonstrated that cilastatin is a megalin inhibitor in the proximal tubule and therefore could represent an attractive alternative to amino acids for this purpose.

**Lessons:**

This case report is a description of a nephroprotective strategy in which partial, and transient tubulopathy is induced, in order to decrease proximal absorption of a tubulotoxic molecule. This little known strategy could be used to prevent proximal tubular injury caused by others megalin-mediated nephrotoxicity medication.

## 1 Introduction

Peptide receptor radionuclide therapy (PRRT) with radiolabeled somatostatin analogs is a targeted internal radiotherapy method used to treat tumors expressing somatostatin receptors, such as neuroendocrine tumors. These tumors usually show a high level of somatostatin receptor expression.^[1]^ The radiolabeled somatostatin binds to these receptors, and the receptor–ligand complex is internalized into the tumor cell.^[2]^ The radionuclide attached to the somatostatin then causes a prolonged and localized irradiation. PRRT has shown to inhibit tumor growth and improve patient symptoms as well as quality of life.[^3^,^4^]

The kidney is the limiting organ to this therapy since proximal tubular cells intake radiolabeled somatostatin^[5]^ causing acute kidney injury, and ultimately end stage renal disease.[^6^,^7^] In proximal tubular cells, the megalin-receptor plays a central role in receptor-mediated endocytosis of somatostatin as well amino acids.[^8^,^9^]

In order to reduce renal toxicity, the co-administration of amino acids during PRRT results in a competition between these 2 megalin receptor ligands, and decreases the uptake of radiolabeled somatostatin.^[10]^ Usually, solutions containing 25 g of lysine and 25 g of arginine are used to inhibit the megalin receptor at the apical pole of the proximal tubular cell.^[11]^

We report here the blood and urinary hydroelectrolytic consequences of an amino acid infusion for [177Lu]-DOTA0-Tyr3-octreotate PRRT premedication.

## 2 Case report

We report the case of a 67-year-old woman with an intestinal neuroendocrine tumor. The diagnosis was made on a lymphadenopathy biopsy in 2011 revealing a neuroendocrine carcinoma without mitosis and a Ki67 <1%. The evolution was marked by a spreading with the appearance of multiple lymphadenopathies and liver metastases in 2015. In 2017, a treatment by [177Lu]-DOTA0-Tyr3-octreotate was proposed.

The patient's weight was 45 kg for 1.60 m with a body surface of 1.41 m^2^. Her general state was preserved: WHO1 and Karnofsky index was 80%. The patient displayed a carcinoid syndrome with flushes including facial erythrosis and a paresthesia. Clinical examination was otherwise normal.

The [177Lu]-DOTA0-Tyr3-octreotate protocol in our center is available in Table 1. In France, amino acid solutions containing 25 g of Lysine and 25 g of Arginine are not available. Therefore, we used a solution of PRIMENE 10% 2 L, which contains 20 amino acids including 22 g/L Lysine and 16.8 g/L Arginine. Blood and urine samples were drawn on the day before, 2 hours after the start of the amino acid treatment, and at the end of the treatment. Patient has provided informed consent for publication of the case.

**Table 1:**
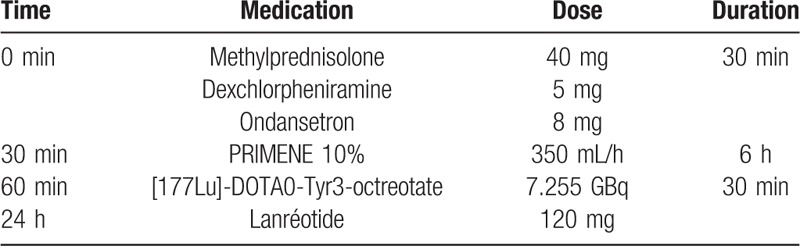
Metabolic radiotherapy by [177Lu]-DOTA0-Tyr3-octreotate protocol.

This protocol was administered to the patient for the first time in February 2017. Two hours after initiation, the patient had presented 7 episodes of vomiting controlled by metoclopramide administration. At the same time, the patient complained of severe cramps. The blood sample showed hypophosphatemia at 0.3 mmol/L justifying intravenous phosphate supplementation. The cramps disappeared after infusion. No hemodynamic instability nor abdominal pain was observed, and the electrocardiogram remained normal throughout the treatment.

Biological data are available in Table 2. We found interesting variation of some specific parameters. Indeed, blood urea increased up to 9.83 mmol/L, hypophosphatemia appeared, and bicarbonate levels decreased reflecting metabolic acidosis. Unfortunately, blood pH measurements were not available. There was no evidence of dyskalemia nor acute kidney injury.

**Table 2:**
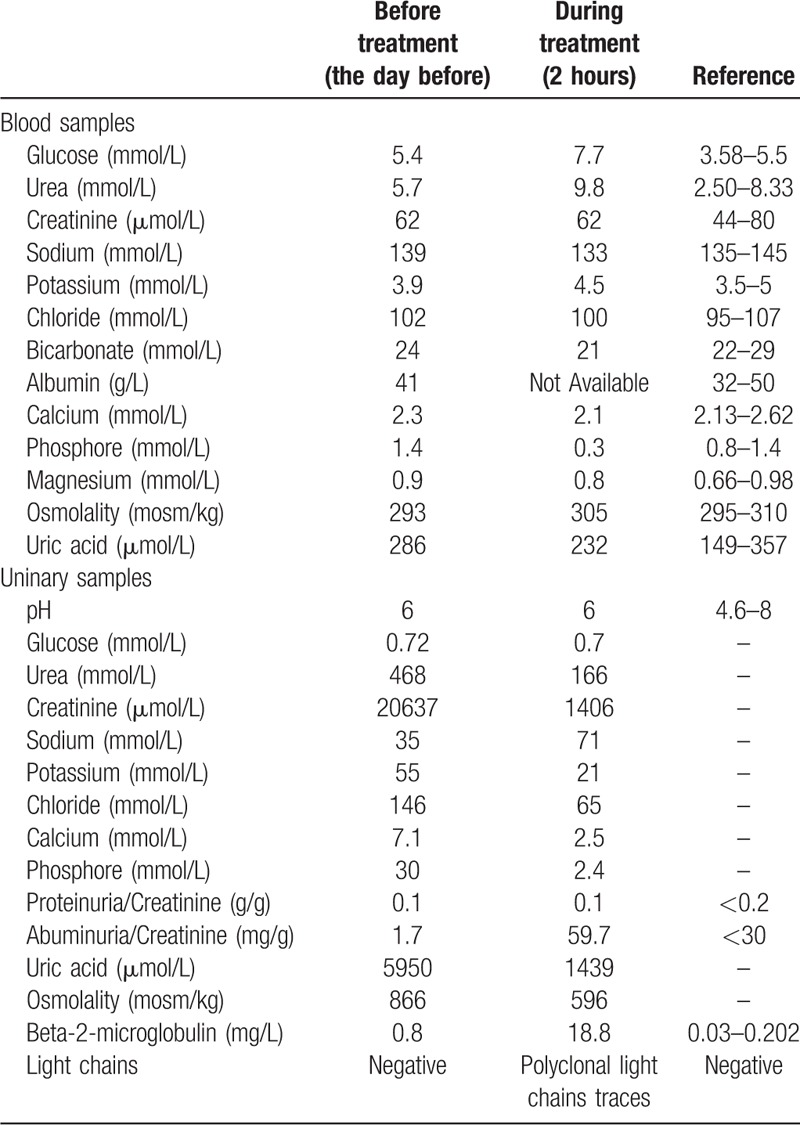
Biologic findings before and during the treatment by [177Lu]-DOTA0-Tyr3-octreotate and amino acids.

As expected, we observed the appearance of a tubular proteinuria containing albumin, β2-microglobulin, urinary polyclonal light chains, transferrin, and lysozymes. A pathological modification of the uric acid excretion fraction was observed, increasing from 6% pre procedure, to 26% per procedure. Likewise, the reabsorption rate of phosphates increased, the fractional tubular reabsorption of phosphate (TRP) going from 93% to 64%. TmPO4/GFR decreased to 0.2. Generalized aminoaciduria related to amino acid infusion was confirmed. We did not find any evidence of glycosuria.

### 2.1 Blood and urine biology normalized on the day after treatment

During and after 4 sessions of metabolic radiotherapy, the patient's creatinine remained stable with no acute kidney injury nor chronic kidney disease.

## 3 Discussion

We describe here a transient and incomplete proximal tubulopathy induced by amino acid infusion in the setting of a first session of metabolic radiotherapy by [177Lu]-DOTA0-Tyr3-octreotate. This tubulopathy is related to the main ligands of megalin^[12]^ which is shown by the appearance of tubular proteinuria and increase in β2-microglobulinuria. The increase in the excretion fraction of uric acid is consistent with this finding. These changes appear to be the consequence of megalin receptor saturation by the infusion of amino acids which reduce the uptake of [177Lu]-DOTA0-Tyr3-octreotate by proximal tubular cells, decrease renal irradiation, and therefore the risk of renal failure.^[6]^ The metabolic pathway of megalin has been shown to be the primary determinant of somatostatin metabolic radiotherapy's nephrotoxicity.^[9]^

There was also a change in the phosphate metabolism with a decrease in TmPO4/GFR indicating a renal phosphate leak resulting in symptomatic hypophosphatemia (cramps). It has also been shown that amino acid infusions are capable of inducing intracellular transfer of phosphates^[13]^ adding to the hypophosphatemia caused by renal loss. Nevertheless megalin endocytosis and phosphate reabsorption are independent pathways, and this renal leak seems to bear witness of a non-specific tubular blockade. The increase in blood urea levels indicate that arginine is metabolized in the urea cycle.

Patient-reported vomiting is a classic side effect of amino acid therapy by relaxation of the lower sphincter of the esophagus.^[14]^ Several physiopathological hypotheses have been suggested by Barone et al, such as the production of nitric oxide or the direct effect of arginine on the central nervous system.^[15]^ The direct effect of aromatic amino acids on gastric parietal cells could also be the cause. Therefore, amino acid infusion prevents renal damage, although severe biological and clinical side effects were noted.

Hori et al recently demonstrated that cilastatin has the ability to inhibit megalin receptors in the proximal tubule.^[16]^ Cilastatin is a reversible inhibitor of renal dihydropeptidases-I^[17]^ and organic anion transporter 3.^[18]^ It is currently available in clinical practice, and is present in the antibiotic imipenem/cilastatin in order to prevent imipenem degradation by dihydropeptidases, and also reduce imipenem tubular damage.^[17]^

Prevention of nephrotoxicity by megalin receptor saturation is a nephroprotective strategy that appears to have interesting results in nuclear medicine by reducing the incidence of renal failure in somatostatin metabolic radiotherapy.^[19]^ Digestive tolerance remains poor, and cilastatin could be an attractive future alternative.

This case report represents a description of a nephroprotective strategy in which the induction of a transient and incomplete tubulopathy decreases the absorption and thus the nephrotoxicity of a tubulotoxic molecule. This strategy could be applied to other molecules whose nephrotoxicity is mediated by megalin as suggested by Hori et al.

The main limitation of this case report is the absence of measurement of the glomerular filtration rate during the radiotherapy session. Also, the large volume of amino acid infused could be sufficient to increase the tubular flow rate here observed.

## 4 Conclusion

We describe an incomplete nephroprotective tubulopathy induced by amino acid infusion in the context of metabolic radiotherapy by [177Lu]-DOTA0-Tyr3-octreotate. This nephroprotective strategy employed by nuclear physicians is not well known by nephrologists, and requires close collaboration between the 2 specialties. Cilastatin could represent an attractive alternative to amino acids in the future.

## Author contributions

**Conceptualization:** Rémi Lenain, Aghilès Hamroun, Georges Lion, Linh Bui, Marc Hazzan, François Provôt.

**Data curation:** Rémi Lenain.

**Supervision:** François Provôt.

**Writing – original draft:** Rémi Lenain.

**Writing – review & editing:** Rémi Lenain, Aghilès Hamroun, Georges Lion, Paul Chamley, Linh Bui, Arnaud Lionet, Marc Hazzan, François Provôt.
